# Single-Step Synthesis of Nanocrystalline Fe-Ni/Fe-Co-Ni Magnetic Alloy Coating via Directional Plasma Spray

**DOI:** 10.3390/ma16072544

**Published:** 2023-03-23

**Authors:** Bo Shi, Chen Li, Ruoyu Han, Qifan Li, Pengfei Li, Xi Chen

**Affiliations:** 1 State Key Laboratory of Mechatronics Engineering and Control, Beijing Institute of Technology, Beijing 100081, China; shibo207@163.com (B.S.); r.han@bit.edu.cn (R.H.); pfli@bit.edu.cn (P.L.); 2 School of Physics, Beijing Institute of Technology, Beijing 100081, China; c.li2018@foxmail.com; 3 School of Materials and Energy, University of Electronic Science and Technology of China, Chengdu 610054, China; li.qifan@uestc.edu.cn

**Keywords:** Fe-Ni-based magnetic alloy thin film, magnetic properties, directional electrical explosion spray, intertwined explosion

## Abstract

Fe-Ni-based nanocrystalline coatings with unique magnetic properties are widely used as soft magnetic materials and usually act as the core component in electronic devices. Nanocrystallized particles and thin films have become a popular contemporary research direction. Electrical explosion, characterized by an ultrafast atomization and quenching rate (d*T*/d*t* ~ 10^9^–10^11^ K/s) for the material, is a unique approach for the rapid “single-step” synthesis of nanomaterials and coatings. In this study, experiments were carried out with intertwined wire under a directional spraying device in atmospheric Ar ambience. Two load systems of Fe-Ni and Fe-Ni-Co were considered in this work. Electrical parameters and high-speed camera images were obtained to reveal the physical mechanism and dynamic process of explosive spraying. The morphologic and crystallographic results were characterized by SEM and XRD. The magnetic properties were measured via VSM equipment, and the parameters of saturation magnetization *M*_s_, residual magnetization *M*_r_, and coercivity *H*_c_ were emphasized in the hysteresis loop pattern. The experimental results indicate that a dense coating was prepared with extremely low porosity, and the morphology of the coating surface shows different regions characterized by solidified chunks and loose particles. XRD patterns showed that crystalline structures were discrepant under two load systems with different Ni weight proportions. Magnetic measurements gave a thin and narrow hysteresis loop, which represents loops with good soft magnetic properties. Quantitatively, coercivity *H*_c_ decreased from 59.3 to 52.6 and from 121.0 to 49.9 for the coatings not containing and containing Co under parallel and perpendicular fields, respectively.

## 1. Introduction

Soft magnetic material refers to the ability to obtain a large magnetization response under a small alternating magnetic field and is the core component in magnetic devices. Fe-Ni-based magnetic materials, typically as permalloys, are among of the most essential soft magnetic materials, characterized by relatively large anisotropic magnetoresistance (AMR) [1], high permeability [2], and small coercivity [3], and have been used as magnetic field sensors [4] and high-sensitivity magnetic heads [5]. For example, the measured AMR sensor HMC1002 manufactured by the Honeywell company consists of two sets of magnetoresistors, and each magnetoresistor contains a permalloy strip with a barber-pole structure with the conductor on top [6]. Such AMR sensors possess fascinating sensitivity and can detect low-intensity magnetic fields; therefore, they are widely used in sensing weak magnetic fields such as the Earth’s field (~48 μT, horizontal component ~19.5 μT in Central Europe) [7]. Magnetoresistive sensors based on the precise acquirement of geomagnetic information can be used in the fields of missile guidance/attitude angle control [8] and compass applications [9,10].

Traditional bulk magnetic materials were always limited in their practical application, especially in high-frequency and microwave fields, because of their low resistivity. Since the rise of the nanomaterials’ field, attention has been drawn to the thin films or fine powders of magnetic materials. Later studies verified that nano-sized magnets (such as nanoparticles or nano-films) still reserve their outstanding properties and preserves the soft magnetic properties, while more attractive effects and satisfactory performance were revealed, such as the giant magnetoresistance (GMR) effect [11,12], which provided better adaption and improvement in practical applications. With the nanocrystallization of magnets, scholars found that the grain size had prominent influence on the magnetic properties [13,14]. For example, Seet et al. developed a high-permeability nanocrystalline permalloy by means of electrodeposition with the sample grain size being several tens of nanometers, and the results showed that the coercivity decreased rapidly and the magnetoimpedance ratio increased greatly with a decreased grain size from 52 to 11 nm [2]. Furthermore, to further improve magnetic performance, such as higher saturation magnetization and lower coercivity, third elements (such as Co, Ag, Cu, etc.) can be added to the raw permalloy [15,16]. Accurately adjusting and controlling the critical elements of the component, microstructure, grain size, etc., are doubtless significant challenges in optimizing the preparation of equipment and methods.

Electrical explosion of wires (EEW) refers to when a pulse current passes through the conductor (usually metal wire or foil), rapid phase change occurs due to Joule heating, and the conductor undergoes liquefication, vaporization, and, finally, ionization into plasma. The extremely high temperature increase rate (d*T*/d*t* ~ 10^11^ K/s), quenching rate (~10^10^ K/s), as well as the intense mechanical effect (shock wave) during an electrical explosion make this method a powerful tool to prepare high-quality fine powders and spray coatings [17,18,19]. Compared with common additive manufacturing methods based on lasers, arcs, or sputtering, which often need complex electrical and control systems and a relatively long preparation time, the EEW single-step method requires very simple equipment to produce samples within the microsecond to millisecond timescale. On one hand, EEW can occur in every material with good conductivity and can drive multi-wire simultaneous explosions; therefore, it can be used to synthesize alloy powders or coatings and can be used to accurately control the component of the final sample [18,20]. On the other hand, the grain size produced during EEW can also be conveniently adjusted by controlling the initial stored energy in the system [21]. Due to the extremely high reaction temperature and quenching rate, satisfactory atomization and sufficient mixing of different elements can be achieved in alloy material synthesis. A special dynamic process of spraying will lead to graded element distribution in alloy coating, which expects to bring novel properties for functional materials preparation [19]. Based on the above advantages, EEW may become a promising method to prepare high-quality nanocrystalline permalloy magnets. 

In this study, an investigation was performed on the permalloy coating prepared by exploding an intertwined multi-wire load via pulsed discharge using a designed directional spraying device. Experiments were implemented with two systems, namely, Fe-Ni-Co and Fe-Ni alloy coatings. Electrical parameters and spatio-temporal images were investigated to analyze the spraying dynamic process. Coating morphology was observed by SEM for both systems, and the crystallographic characteristics were analyzed by XRD for the synthesized coatings with different Ni weight proportions. The formation mechanism was primarily discussed based on the above results. Finally, magnetic properties were measured by VSM and analyzed through profiling the hysteresis loops.

## 2. Experimental Setup

### 2.1. Materials Preparation and Parameters Measurement

**Figure 1:**
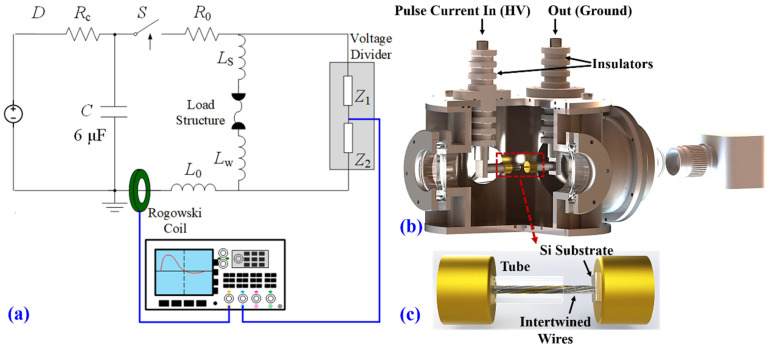
Schematics of the experimental setup and configurations: (**a**) circuit diagram; (**b**) cross-sectional view of the chamber; and (**c**) detailed drawing of the directional spraying device.

The experiments were conducted using a microsecond-timescale pulsed current source based on a 6 µF pulse capacitor and a triggered spark gap. The circuit is shown in Figure 1a, and the DC power supply charged the capacitor up to 12.9 kV (about 500 J stored energy). Once the spark gap was triggered, the pulsed current passed through the load and drove an electrical explosion inside the discharge chamber. The schematic diagram of the chamber is illustrated in Figure 1b. A detailed drawing of the exploding load structure, namely, the directional spraying device, is shown in Figure 1c. More details about the experimental setup can be found in [19,22,23]. The device was composed of two copper electrodes, a semi-closed tube, an intertwined wire, and a silicon substrate. Specifically, the intertwined wire was made from several independent metal wires with identical lengths twining with each other. When the explosion occurred, the high-speed metal jet sprayed out and formed an alloy coating on the silicon substrate. 

The discharge voltage *u* and current *i* were measured with a PVM-5 probe (bandwidth of 80 MHz) and a Rogowski coil (bandwidth of 4 MHz), respectively. The resistive load voltage *u*_R_ was estimated as

(1) $$u_{R}(t)=u-L_{s}\frac{di}{dt}$$

where *L*_s_ refers to the inductance of the wire and its holder. The correctness of voltage and current measurement was examined by conducting the pulsed current with a low-inductance (nH level) ceramic resistor [22,23]. The energy deposition *E*_d_ was calculated as

(2) $$E_{d}=\int u_{R}(i)\cdot i(t)dt$$

High-speed cameras (Phantom VEO) were used to record time-resolved images of the explosion.

### 2.2. Characterization

Morphologic and crystallographic characterizations included SEM (Zeiss, Jena, Germany) and XRD (Rigaku, Tokyo, Japan, Ultima IV), respectively. The XRD pattern of the sample was obtained using Cu Kα radiation (λ = 0.15406 nm) at the working voltage and current of 40 kV and 40 mA, and the recorded range at 2θ was from 30 to 80° with a step size of 0.02°. Magnetic performance was detected by the vibrating sample magnetometer (VSM-LakeShore 8604), and two magnetic field directions, namely, parallel and perpendicular to the coating surface, were applied to measure the magnetic properties of the samples. Furthermore, experiments were conducted in Ar gas ambience, and the prepared samples were stored in a vacuum chamber to avoid possible oxidation.

### 2.3. Experiment Designs

Two experimental methods were used in this paper. System 1 involved an explosion of an intertwined wire made from Fe, Ni, and Co metal wires. System 2 involved an explosion of an intertwined wire made from Fe and Ni metal wires with different weight proportions. The coating composition was selected by changing the diameter of the Ni wire. In this paper, system 1 was mainly investigated with regard to electrical characteristics, dynamic process, and crystallographic results; meanwhile, system 2 was used as a comparison for the synthesized alloy coating. The detailed load parameters are listed in Table 1. Furthermore, the semi-closed tube had an inner diameter of 8 mm and a length of 2 cm. A monocrystalline silicon was used as the substrate to deposit the coating. Experiments were performed in atmospheric Ar ambience for both systems.

## 3. Results and Discussion

### 3.1. Electrical Characteristics and Dynamic Process

**Figure 2:**
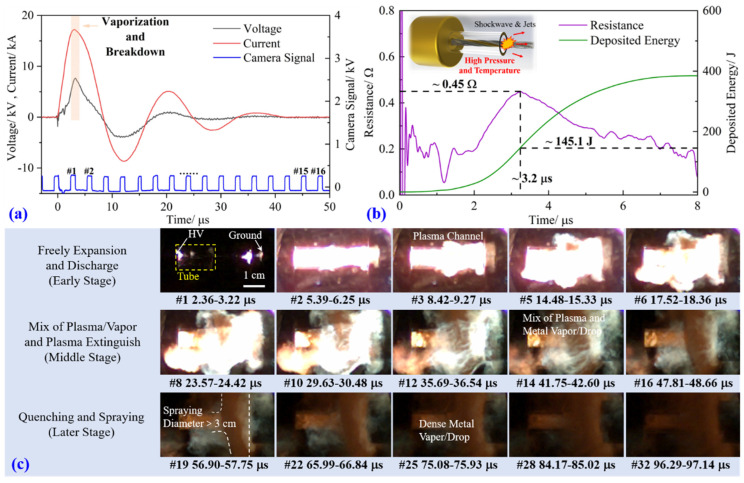
Intertwined wire explosion made of Fe, Co, and Ni under the directional spraying device: (**a**) current and voltage waveforms; (**b**) resistance and deposited energy waveforms; (**c**) high-speed (self-emission) images.

In the conditions of the joint explosion of multi-species metal wires, the electrical characteristics and dynamics behaviors are usually complex and display asynchronicity [19,24]. To prepare the alloy coating, the intertwined wire explosion experiment with Fe, Ni, and Co metal wires using a directional spraying device was implemented, and the physical process and mechanism were analyzed. The main results are shown in Figure 2. Figure 2a,b presents the basic electrical parameters: at approximately 3.1 μs, the current reaches the peak value of ~17.3 kA, and then it undergoes a mild decrease for about 1.3 μs, and, finally, a rapid decrease and oscillation. This period signifies the vaporization and breakdown processes during the explosion; meanwhile, the voltage reaches its peak because of the drastically increased resistance. At approximately 3.2 μs, the peak value of resistance is ~0.45 Ω, and then it begins to decrease due to the formation of relatively low-resistivity plasma, reducing the whole channel’s resistance. At the moment of the resistance peak, the deposited energy is ~145.1 J, which is less than the indispensable energy for the full vaporization of the intertwined wire 290.6 J; therefore, the system should be a mixture of major metal vapor and some un-vaporized drops, and this can be verified by the high-speed images. Observing the high-speed images in Figure 2c, the whole discharge can be divided into three stages. At the early stage, the wire explosion process is identical in free air before the discharge channel arrives at the tube wall. Then, the channel is restrained by the tube, high-density materials gather in the tube, and the remaining part outside the tube expands rapidly; as a result, breakdown occurs in this low-density part. The whole discharge channel is a mix of low-density ionized plasma and high-density un-ionized metal vapor and drops, visible in frames #12 and #14. At 47.8 μs (frame #16), the discharge ends, and plasma extinguishes gradually with dense metal vapor/drops remaining in the column. From 56.9 μs, shown in frame #19, the jet profile resembles a funnel, and the high-density materials are propelled continuously toward the Si substrate. The design of the directional spraying device allows for the spraying diameter larger than 3 cm.

### 3.2. Morphologic and Crystallographic Characterizations

**Figure 3:**
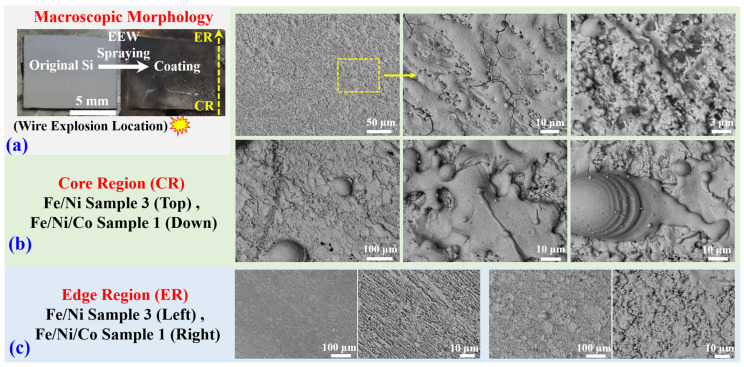
Si substrate after spraying: (**a**) macroscopic morphology; (**b**) microcosmic SEM images of both samples in the CR; (**c**) microscopic SEM images of both two systems in the ER.

Figure 3 shows the morphological results of the sprayed coating. In this experiment, sample 3 of Fe-Ni and sample 1 of Fe-Ni-Co are studied. Figure 3a shows the macroscopic morphology of the coating typically formed on the monocrystalline Si substrate during the EEW process. The wires explosion is located at one side of the substrate, and the coating area close to the explosion is called the core region (CR), while the area further away from the explosion is called the edge region (ER). The CR region has a silvery metal luster, while the ER is black. Figure 3b presents the SEM images of the CR region of the two systems. At a low magnification of the CR, the coating surface is very dense with extremely low porosity. For sample 3, the surface is relatively flat, while sample 1 has a rough surface with some solidified chunks with sizes of several tens of micrometers. The higher magnification shows the solidified state of the coating surface for both samples. During the explosion and spraying process, un-vaporized liquid drops with a high spraying speed and large mass are produced. The liquid drops experience cooling, and, finally, form the main coating in the CR region. The drops are most prevalent for large-mass loads at a fixed initial stored energy, as in sample 1. The residual parts of deposited material such as fine spherical particles are visible on the surface of the solidified chunks in the last photograph in the top row and the last two images in the bottom row of Figure 3b. Those particles are created from the hot metal vapors after the quenching, nucleation, and deposition process on the coating surface. The images of ER areas, as shown in Figure 3c, show that the coating surface is relatively loose, and the formation of newly produced particles seems to be dominant in this region.

**Figure 4:**
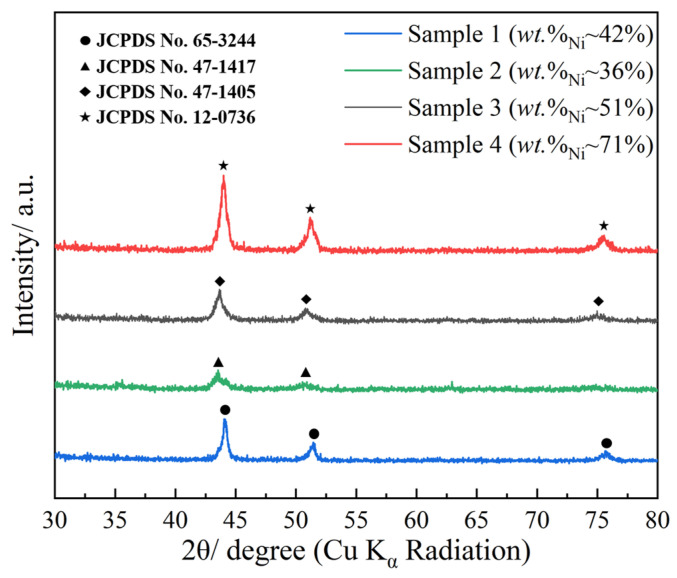
XRD patterns of samples 1 to 4.

Crystallographic characterization for all four samples was performed by XRD and is shown in Figure 4. The characteristic diffraction peaks appearing at approximately 2θ = 44.0, 51.2, and 75.5° correspond to the (111), (200), (220) planes of the face-centered cubic (fcc) phase Fe-Ni alloy, respectively. The XRD pattern of Fe-Ni-Co alloy coating is also similar to that of the Fe-Ni system. Positions of diffraction peaks of samples 1 to 4 correspond well with the standard card of JCPDS No. 65-3244, 47-1417, 47-1405, and 12-0736, respectively. According to Bragg’s law, *nλ* = *d*2sin*θ*, and Scherrer’s law, D = *kλ*/*B*cos*θ*, the crystal lattice distance d and grain size D can be calculated, where *λ* is the waveform of the Cu target (0.154 nm), *k* is the Scherrer constant (0.89), *B* is the FWHM of the diffraction peak, and *θ* is the degree of the diffraction peak. Detailed parameters are given in Table 2. For different samples, the main peak experiences a slight shift, illustrating that the crystalline structure and component are different, which can be verified by the changed crystal lattice distance *d* and the calculated cell constant [25]. In addition, for samples 2 to 4, the peaks become weaker and broader when reducing the weight proportion of Ni; widening of the FWHM results in smaller grain size, demonstrating decreased crystallinity and a more amorphous nature. This phenomenon can be explained by the fact that, under fixed initial stored energy, a lower load mass provides a higher proportion of hot metal vapor; as a result, vapors undergo quenching, nucleation, and, finally, recrystallization, producing large amounts of nanocrystalline and amorphous material.

### 3.3. Magnetic Property 

**Figure 5:**
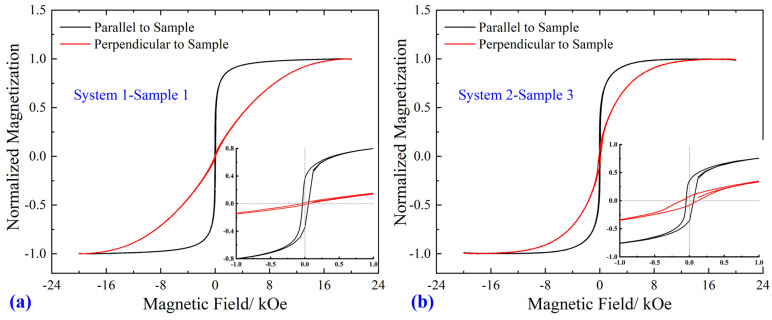
Hysteresis loops of different samples measured by VSM at room temperature: (**a**) system 1–sample 1; (**b**) system 2–sample 3. The insets in (**a**,**b**) are the partial enlarged patterns at magnetic field intensity from −1.0 to 1.0 kOe.

The magnetic properties of the prepared alloy coating were measured via VSM at room temperature, as shown in Figure 5. For the two considered systems, namely, Fe-Ni-Co (Sample 1) and Fe-Ni (Sample 3), the shape of the measured hysteresis loops was thin and narrow, presenting good soft magnetic characteristics. An external magnetic field was applied both parallel and perpendicular to the samples. The magnetic films show magnetic anisotropy, manifested as the discrepant hysteresis loops. For both samples 1 and 3, the saturation magnetization *M*_s_ saturates faster under the parallel field, indicating easy axis magnetization in the coating plane. To show the characteristics of coercivity *H*_c_, partial enlarged patterns of magnetic field intensity from −1.0 to 1.0 kOe are presented as the insets in Figure 5a,b. For sample 3, the coercivity *H*_c_ is 59.3 and 121.0 Oe under the parallel and perpendicular field, respectively. When measuring sample 1, the *H*_c_ is the same, which is approximately 49.9 and 52.6 Oe under the two fields. Moreover, adding Co into the permalloy coating decreases the *H*_c_, which is satisfactory for soft magnetic materials.

Coercivity is one of the most critical parameters that can represent the magnetic properties of soft magnetic materials. Common Fe-Ni-based magnetic film preparation methods, such as thermal evaporation, electrochemically deposited magnetron sputtering can synthesize film with the *H*_c_ several tens to hundreds of Oe [15,16,26]. In comparison, our Fe-Ni and Fe-Ni-Co films possess the same orders of magnitude of *H*_c_, which means the synthesized films by the EEW method are feasible and applicable. However, some studies indicate that the coercivity could reach less than 10 Oe, and the magnetic properties were influenced by numerous factors, such as film thickness, grain size, added elements, substrate materials, etc. [27,28]. Deeper investigation will proceed in the future work.

## 4. Conclusions

The behaviors of the electrical explosion spraying of intertwined wire using a directional spraying device in atmospheric Ar ambience were investigated. Spatio-temporal images showed that the effective diameter of the funnel-like spraying area was, more or less, 3 cm. The morphology and crystalline structure of the two synthesized systems of Fe-Ni and Fe-Ni-Co coatings were measured. Dense coatings with a low porosity were prepared. For both systems, the morphologic characteristics showed discrepancies in different regions, manifested as solidified chunks in CR and loose particles in ER. XRD patterns indicated that the Ni weight proportion in the coating influenced the crystalline structure of the sample. Thin and narrow hysteresis loops were obtained for both systems, especially under the parallel magnetic field, which represented relatively good soft magnetic properties. The changes between parallel and perpendicular coercivity were 49.9 and 52.6 for sample 1 and 59.3 and 121 for sample 3, respectively. A nanocrystalline permalloy film with relatively good soft magnetic properties was successfully prepared via the method of directional electrical explosion spraying.

## Figures and Tables

**Table 1:** Detailed load parameters used in experiments.

Serials		Constituent Wires	Wires Specification		Weight Proportion of Ni
			Diameter	Length	
System 1	Sample 1	Fe, Ni, Co	Fe-200 μm, Ni-300 μm Co-300 μm	4 cm	~42%
System 2	Sample 2	Fe, Ni	Fe-200 μm, Ni-150 μm	4 cm	~36%
	Sample 3		Fe-200 μm, Ni-200 μm		~51%
	Sample 4		Fe-200 μm, Ni-300 μm		~71%

**Table 2:** Detailed value of the diffraction peaks and the calculated parameters.

Sample No.	2θ/(°)			*d* ^1^ _(111)_/nm	*D* ^2^ _(111)_/nm	Cell Constant/Å (a = b = c)
	(111)	(200)	(220)			
1	44.12	51.40	75.65	2.05	17.51	3.5523
2	43.49	50.67	74.53	2.07	10.43	3.5975
3	43.60	50.79	74.67	2.07	14.09	3.5922
4	43.91	51.19	75.44	2.06	13.90	3.5600

^1^ *d* is the crystal lattice distance. ^2^ *D* is the grain size.

## Data Availability

The data presented in this study are available on request from the corresponding author.
